# Supplementary figures and images for: Transcriptional subtypes on immune microenvironment and predicting postoperative recurrence and metastasis in human pheochromocytoma and paraganglioma
Source: eLife. 2025 Dec 16;14:RP107108. doi: 10.7554/eLife.107108 (PMC12707812; doi:10.7554/eLife.107108)

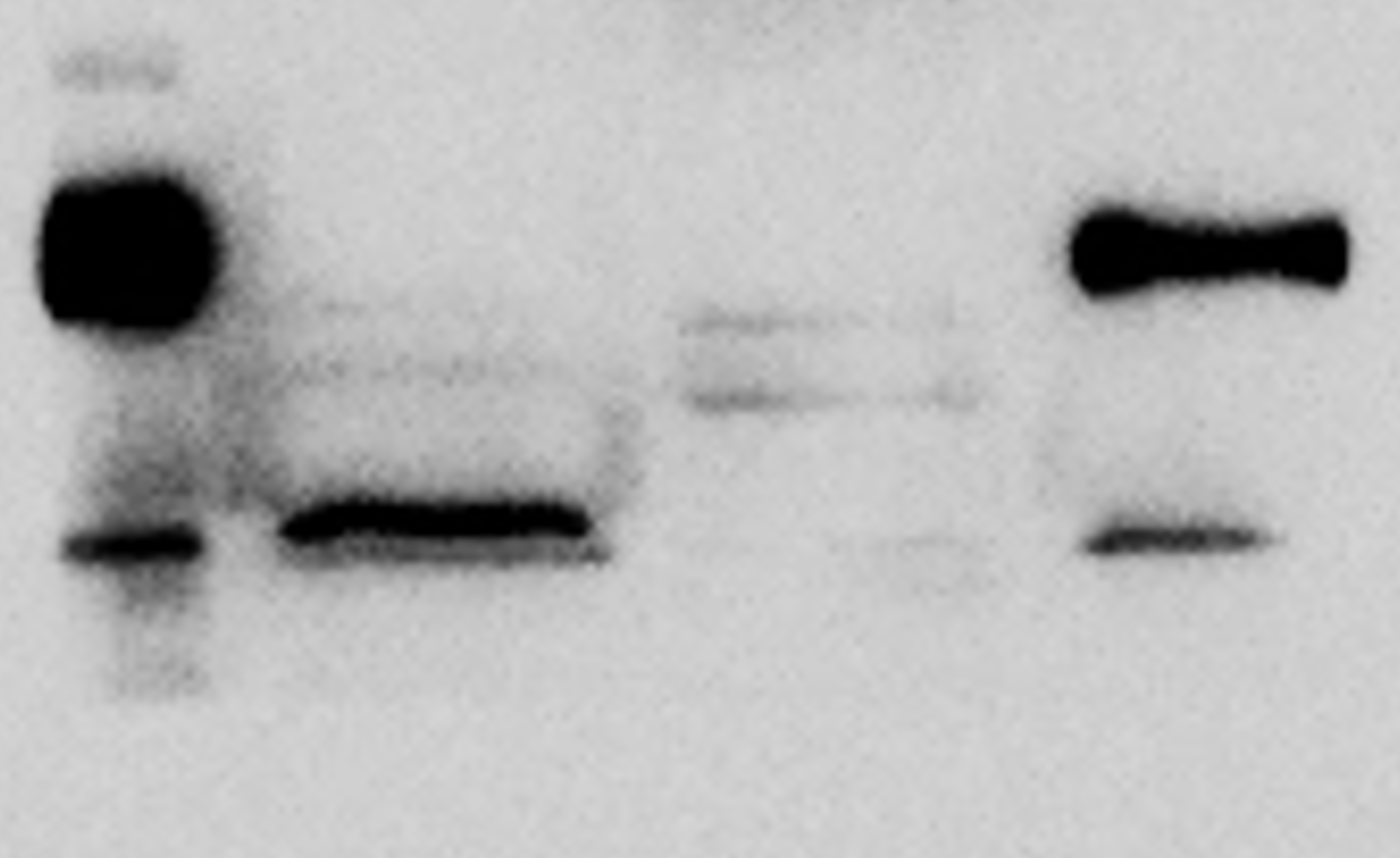

Supplement: Figure 6—source data 1. [file elife-107108-fig6-data1.zip › elife-107108-fig6-data1/ANGPT2.tif]

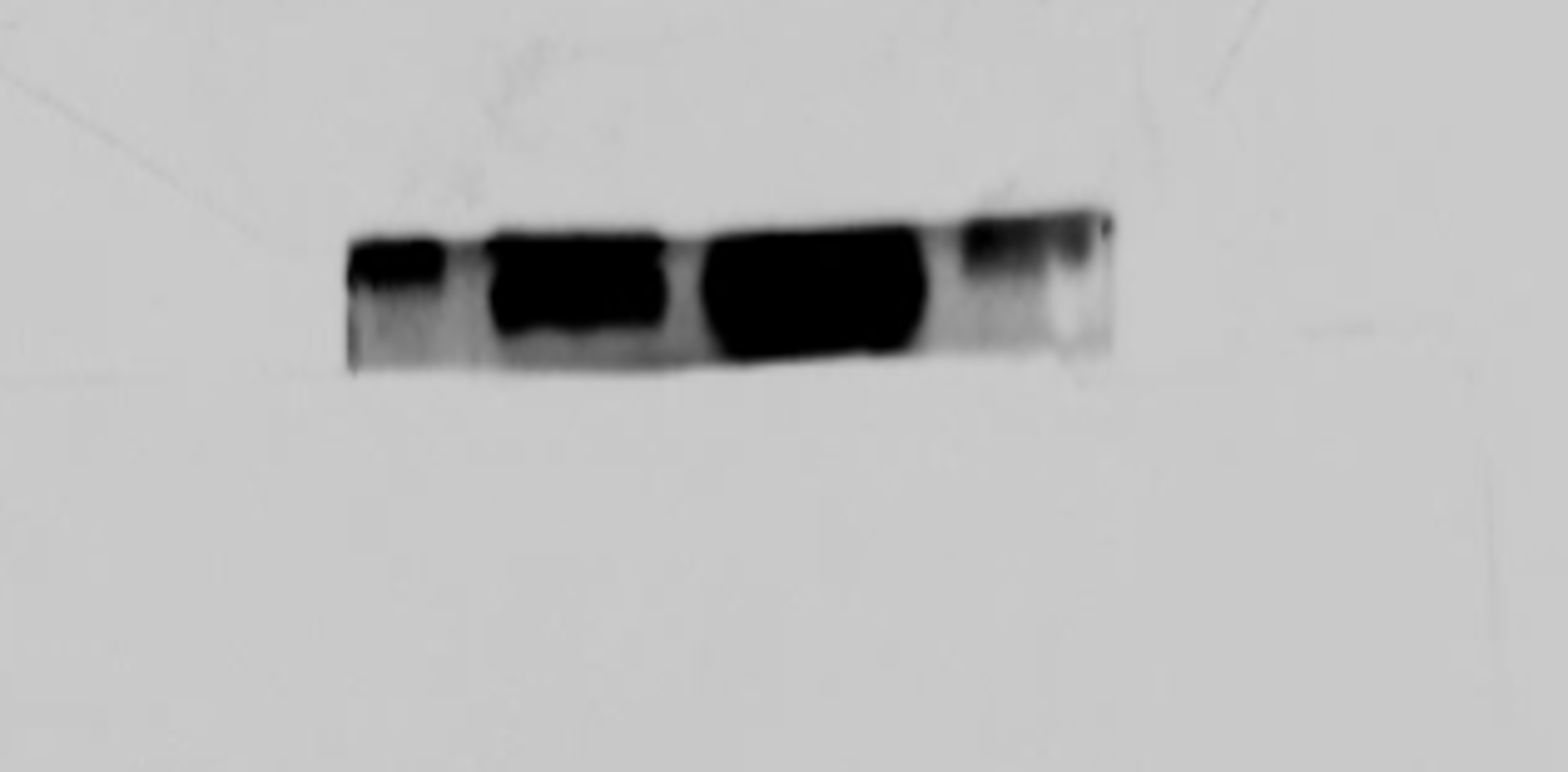

Supplement: Figure 6—source data 1. [file elife-107108-fig6-data1.zip › elife-107108-fig6-data1/a┬-actin.tif]

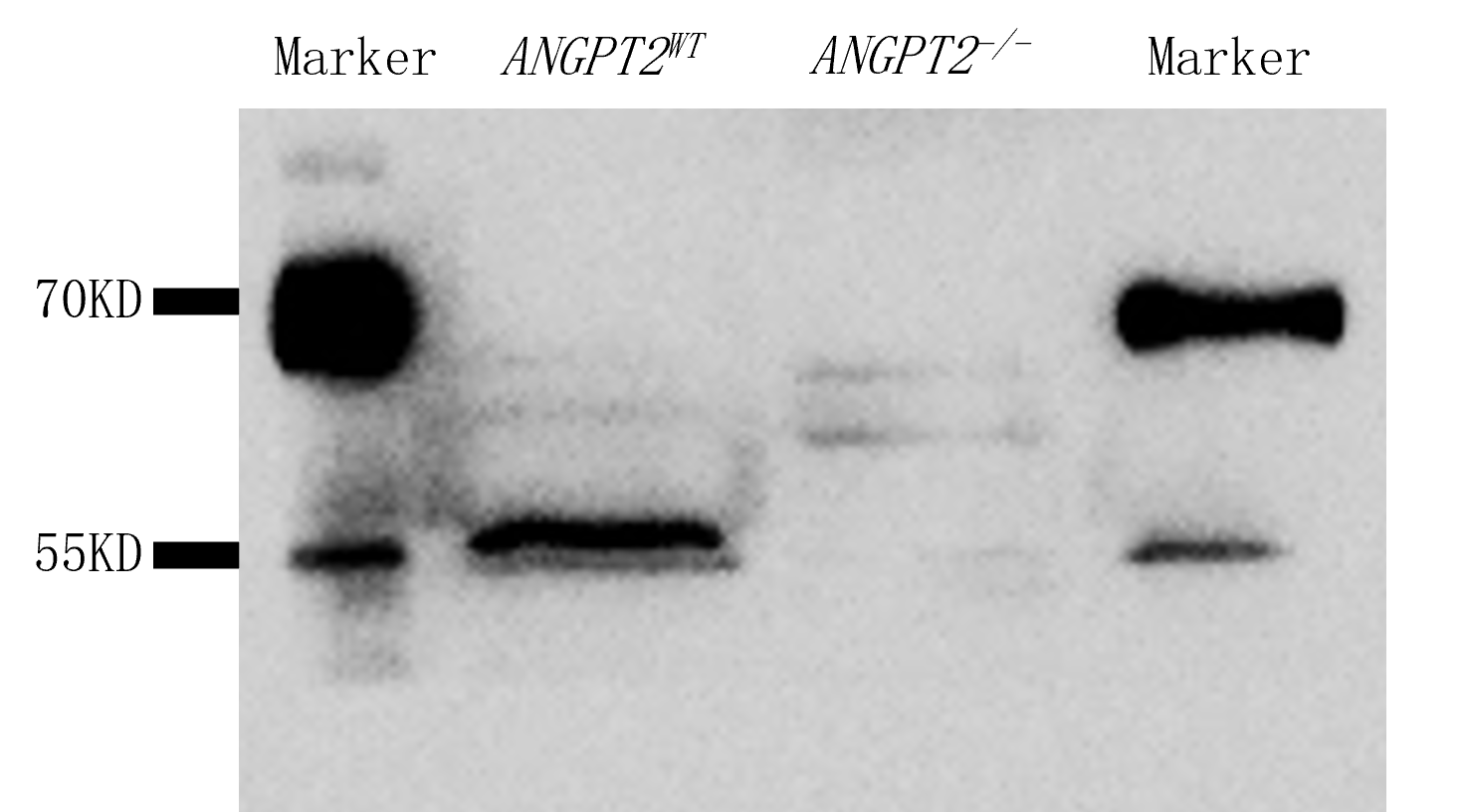

Supplement: Figure 6—source data 2. [file elife-107108-fig6-data2.zip › elife-107108-fig6-data2/ANGPT2-M.tif]

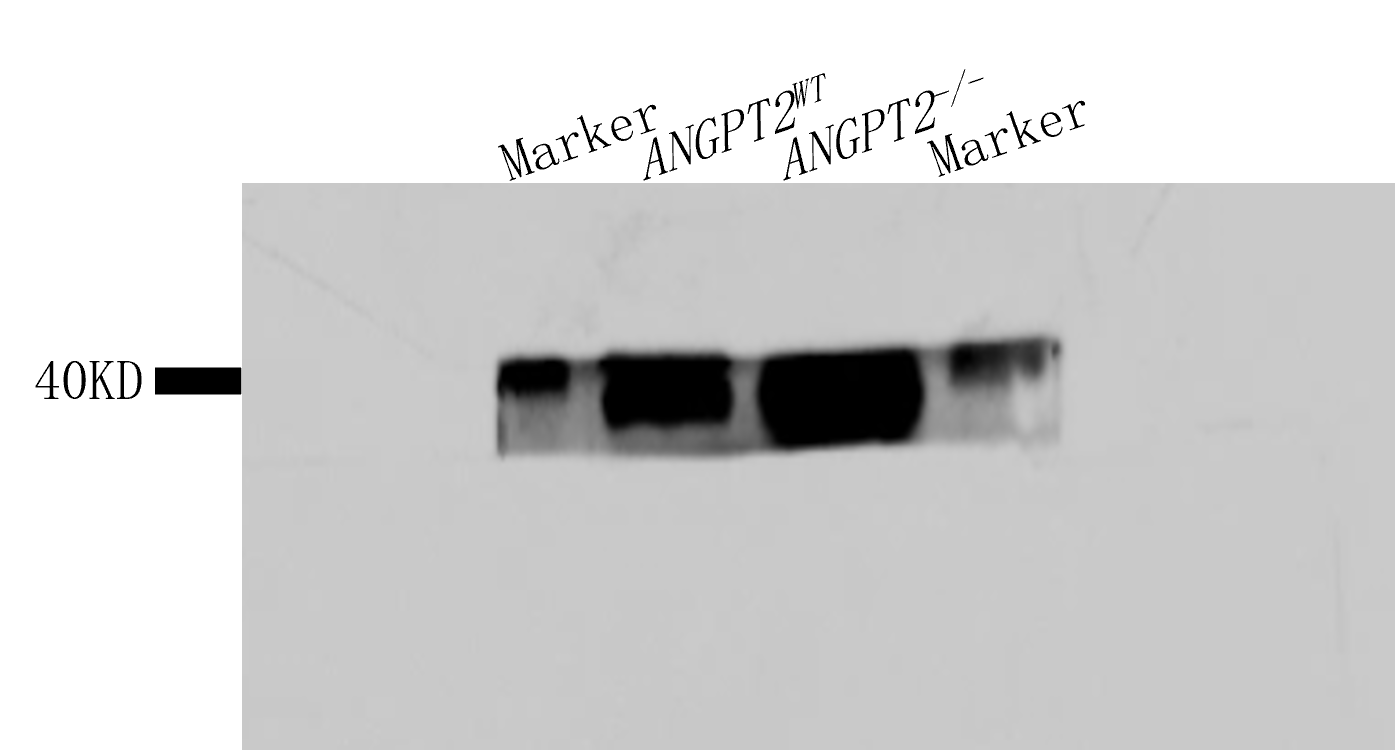

Supplement: Figure 6—source data 2. [file elife-107108-fig6-data2.zip › elife-107108-fig6-data2/a┬-actin-M.tif]
